# Redundant Fault Diagnosis for Photovoltaic Systems Based on an IRT Low-Cost Sensor

**DOI:** 10.3390/s23031314

**Published:** 2023-01-24

**Authors:** Joan Ochoa, Emilio García, Eduardo Quiles, Antonio Correcher

**Affiliations:** Instituto de Automática e Informática Industrial, Universitat Politècnica de València, Camino de Vera, s/n, 46022 Valencia, Spain

**Keywords:** PV modules, PV plants, predictive fault diagnosis, IRT sensors, hot-spot failures

## Abstract

In large solar farms, supervision is an exhaustive task, often carried out manually by field technicians. Over time, automated or semi-automated fault detection and prevention methods in large photovoltaic plants are becoming increasingly common. The same does not apply when talking about small or medium-sized installations, where the cost of supervision at such level would mean total economic infeasibility. Although there are prevention protocols by suppliers, periodic inspections of the facilities by technicians do not ensure that faults such as the appearance of hot-spots are detected in time. That is why, nowadays, the only way of continuous supervision of a small or medium installation is often carried out by unqualified people and in a purely visual way. In this work, the development of a low-cost system prototype is proposed for the supervision of a medium or small photovoltaic installation based on the acquisition and treatment of thermographic images, with the aim of investigating the feasibility of an actual implementation. The work focuses on the system’s ability to detect hot-spots in supervised panels and successfully report detected faults. To achieve this goal, a low-cost thermal imaging camera is used for development, applying common image processing techniques, operating with OpenCV and MATLAB R2021b libraries. In this way, it is possible to demonstrate that it is achievable to successfully detect the hottest points of a photovoltaic (PV) installation with a much cheaper camera than the cameras used in today’s thermographic inspections, opening up the possibilities of creating a fully developed low-cost thermographic surveillance system.

## 1. Introduction

Solar panels are exposed to high degradation due to outdoor operation. Therefore, a good combination of online predictive diagnosis techniques is required to avoid failures leading to interrupting power generation.

Initially, the predictive diagnosis techniques explicitly applied to each solar panel guarantee early detection and isolation of the predictive symptoms that warn of the occurrence of some degrading phenomenon that can lead to the inevitable development of different types of faults or failures [1,2,3]. For example, let us suppose that one panel in the array begins to develop degradation due to shading in an incipient manner. In that case, it will do so as a reduction symptom in power generated by the solar panel [2]. However, this same symptom can be associated with developing other types of faults in the solar panels, such as breakage, discoloration, hot-spots, and potential induced degradation PID. Therefore, to carry out a deeper analysis of the type of fault that occurred, it is convenient to use modern techniques such as thermography on the affected panel. The predictive diagnosis aims to detect and isolate predictive symptoms, acting in advance on the photovoltaic panels by disconnecting them in order to prevent the development of intermittent or permanent failures.

PV modules (PVMs) can experience substantial damages that affect constituent materials such as metals, crystals, encapsulating polymers, and, especially, PV cells. Consequently, the PV plants will decrease their performance in the power generation capacity. It has been shown that power reduction is the best indicator to detect the lack of performance and degradation of a photovoltaic solar installation [4]. For this reason, its study is vital to allow a correct estimation of the energy production, the useful life of the system, and, consequently, the installation’s reliability and financial viability.

Some of the failures that occur in PVMs (for example, hot-spots) are unsolvable when they reach a particular stage of development. Then, the only viable solution to the problem is the total replacement of the PVM. Much research has been carried out to diagnose failures in PV installations. However, in most cases, these studies have focused on analysing the reduction of the power experienced in the inverter of the PVM strings. Studying inverter signals avoids investments in extra equipment for the supervision of the facilities. However, it decreases the diagnosis power over PVMs. Rapid detection and isolation of the large number and diversity of failures in the PVMs need extra sensors to isolate the failures correctly. Objectively and without a doubt, the basic unit capable of receiving predictive maintenance is the PVM, where most of the failures of the installation are unequivocally developed, and the effects of degradation are observable. Consequently, from the point of view of fault diagnosis, it is interesting to consider the detection, isolation, and analysis explicitly applied in each PVM.

Degradation modes are called the process by which PVMs lose their characteristic properties: electrical, optical, chemical, or mechanical. The degradation modes are physically manifested in the PVMs, in some of their elements or several, simultaneously. Moreover, it is difficult to differentiate what has produced the degradation since it can be due to concurrent causes. The root cause of the failure may be due to synergies between different mechanisms influenced, both in the moment of its appearance and severity, by a series of environmental factors [5,6].

Due to its operating principle, a PV installation is subject to the climatic/environmental stress of the place where it is installed, including the following factors:

- Solar radiation: essential for the installation operation to produce the PV effect. It is also detrimental by giving rise to high temperatures in the PVM.
- Ultraviolet radiation: it is the part of the solar spectrum with the highest energy and produces photodegradation when interacting with the PVM; the encapsulant is the most damaged element, producing chemical reactions in the constituent materials.
- Humidity: penetrating the module can affect the electrical connections causing oxidation or corrosion in them, being one of the most aggressive meteorological elements for the photovoltaic module.
- Snow: this element can give rise to static loads that can vary from 30–50 kg/m^3^ for fresh snow and 800–900 kg/m^3^ for frozen snow, causing breakage of the module itself or the installation structure.
- Wind: this factor can affect continuously, giving rise to a static and dynamic load for the PVMs, subjecting them to vibrations that are very detrimental to their structural integrity [7].
- Hail: this element causes breakage due to impacts on the glass or other elements, significantly affecting the module’s performance.
- Temperature changes: temperature changes affect the module producing thermo-mechanical stress. Since the different elements that make up the PVM (glass cover, solar cells, encapsulant, and rear cover) have different Young’s modules and expansion coefficients, stresses can be generated that lead to the breakage of the cells or delamination of encapsulation materials.
- Dust and dirt of different origins in suspension contribute to the shading effect and, in combination with the wind, cause abrasion, damaging the active face of the photovoltaic modules. Bird droppings can produce the same shading and an especially corrosive effect on the active face of the module. Losses due to these factors reach highly variable values. For example, the work presented in [8] shows losses in the range of 16% to 27% without having carried out any cleaning action in 18 years of operation. Adding an anti-dirt layer to the module cover reduces losses due to this factor, compared to an untreated PVM [9].
- Salt: in those installations near the coast or the sea, the salt can cause corrosion on some PVM elements, such as metallic or polymeric elements.
- Gas: in those PV plants located near industrial facilities, some gases (O_3_, NH_3_, SO_2_, NO_2_, H_2_S, Cl_2_...) may appear in the atmosphere, which alone or in combination with humidity can cause corrosion by becoming acids (HNO_3_, HCl, H_2_SO_4_...).
- Electrical discharges: although this environmental factor has not been analyzed in depth, it can also cause degradation in photovoltaic modules [10].
- Acts of vandalism: despite not being a weather factor, can be considered an environmental factor.

The factors above do not usually appear in isolation but rather concurrently, so the degradation of photovoltaic modules increases. Considering environmental factors such as materials that make up a photovoltaic module, we can establish the following degradation modes that can appear in crystalline Si PVMs [11]:

- Delamination: loss of adhesion between the different layers of material that make up the photovoltaic module. It causes two effects: the reflection of light is increased. Therefore, the light that can be used for the photovoltaic effect is lost, the entry of moisture into the PVM is facilitated, and another mode of degradation can be generated: corrosion.
- Discoloration: it is due to the degradation of the encapsulant (compound, as we have seen previously, usually Ethylene Vinyl Acetate (EVA) or of the adhesive material between the glass and the photovoltaic cells. The color changes to yellow (a phenomenon called yellowing in English) and brown (browning in English). In this way, the optical transmission is modified, reducing the energy generated by the photovoltaic module.
- Corrosion: it consists of the destruction of a metal by an electrochemical reaction caused by the environment in which it is found, due to contact with water or humidity. Moisture inside the PVM increases the electrical conductivity of existing metals, causing leakage currents. Corrosion also attacks the adhesion between the cells and the metal edge (usually aluminum), causing delamination and favoring the entry of more moisture. The test based on the IEC 61215 standard [7] produces, among others, this type of degradation mechanism.
- Module breakage: roof glass breakage is a significant degradation factor in photovoltaic modules. They appear in most cases during installation, maintenance, and transport operations where photovoltaic cells are too often subjected to vibrations, which facilitate the appearance of cracks or even breakage [12].
- Bubbles: they bear some similarity to delamination, and it becomes a precursor symptom of the latter’s appearance. The bubbles are formed due to chemical reactions that emit gases trapped inside the photovoltaic module, making it difficult to dissipate heat in the photovoltaic cells, reducing their useful life due to the increase in temperature that occurs.
- Potential Induced Degradation (PID): appears when serial connections are used between different PVMs, producing a sum of hundreds of volts in a string (300–500 V). PID causes a leakage current that passes from the active layer of the silicon through the encapsulant (EVA) and the glass front cover. Then, the current reaches the metal frame, which is favored in hot and humid climates, and in modules that have received an application of an anti-reflective coating (ARC, Anti Reflective Coating) on its surface [13,14].
- Degradation by electrical discharges: PVMs can be subjected to electrical discharges from storms (lightning), which can damage the photovoltaic module either through direct discharge or through the magnetic coupling that occurs. It is necessary to design an adequate protection system to avoid this damage [15].
- Modes of degradation and climatology: Climatology influences the different degradation mechanisms. Delamination and discoloration will appear in areas where ultraviolet radiation is high. Corrosion increases in climates where humidity is an essential factor. The degradation induced by PID is aggravated in this type of climatology [16]. Also, the probability of electric lightning strikes in the chosen location will influence the expected energy generation and dictate specific degradation mechanisms.

Among all the degradation modes described above, the generation of hot-spots in PVMs stands out for their criticality, frequency of appearance, and critical risk of irreversible damage in PV modules. That is because the phenomenon of hot-spots triggers some of the degradation modes mentioned above, such as glass breakage, delamination of the insulating material, lack of electrical insulation, and sometimes catastrophic fires. Before their qualification, to protect the PVMs with a guarantee, there are international standards that aim to carry out resistance tests to the appearance of hot-spots.

The appearance of hot-spots in a PV installation is a problem of great importance since it affects not only the production but also the useful life of installation (it produces premature degradation and aging). Hot-spots have been a well-known phenomenon for over 50 years [17], and it persists today [18,19]. Hot-spots frequently appear when a panel is partially exposed to shaded areas. Consequently, the affected cell (or group of cells) operates in reverse polarization conditions, dissipating energy instead of generating it. Therefore, the generation of very high temperatures favors the appearance of hot-spots, gradually degrading both the generated power and the components of the encapsulation material of the PVM [20]. One of the reasons for the greater frequency of the appearance of hot-spots has been the tendency of PVM manufacturers to use thinner wafers, but less resistant to the appearance of microcracks, with a greater propensity to develop them in the manufacturing, transportation, and even installation phases [20,21]. Reports from the UK have highlighted annual power losses of 18.9% associated with shadowing and persistent inverter failures [22].

However, it has been possible to verify that other causes are the origin of the appearance of hot-spots:

- When mismatching occurs between solar cells [23], that is, there is a mismatch in the electrical characteristics between the interconnected cells.
- When the cell has been damaged [24].
- The degree of uniformity of the incident solar radiation.
- The type of connection between modules.
- The monitoring of the maximum power point that is carried out on the solar controller.

Under these operating conditions, the cell can present points where its temperature rises in relation to others surrounding it. Heat dissipation can cause overheating and, in severe cases of high temperature, cause melting of welds, irreversible damage to the encapsulating material, and sometimes cause dangerous fires. These areas with a temperature significantly higher than the rest of the module are called hot-spots. In investigations on the appearance of hot-spots in two large PV plants connected to the grid, it was detected after a visual inspection of trackers that energy production decreased at an abnormal rate. The detected hot-spots appeared both in the solar cells and in the resistive solder bonds (RSB) between the cells and the contact ribbons. Both types caused similar irreversible damage to the photovoltaic modules, but the latter was found to be primarily responsible for the decrease in energy production detected. The results of this research were of interest for the routine maintenance of large photovoltaic plants connected to the grid [25].

In PV installations, when hot-spots are detected, the PVM should be taken out of service to favor the analysis of the root cause of the problem. If the PVM is not disconnected, it can be unrecoverable in less than a day. Therefore, in our opinion, the state of the facility’s condition, the early detection, and the isolation of the degradation phenomenon should be adjusted to maintain adequate sampling frequencies for the mentioned degradation development time intervals.

It is difficult for the human eye to detect hot-spots, especially in the initial phase of its development. A technology currently being implemented in the diagnosis and condition monitoring of similar problems is infrared (IR) thermography [26]. IR cameras can be used as surface temperature sensors for indoor or outdoor applications. Passive thermography applications monitor the temperature of a surface under working conditions. More recently, active thermography studies the effects of external energy sources on surfaces. An external heat source introduces a heat perturbation, and the IR camera records a sequence of the temperature variation due to defects. A considerable amount of non-destructive test procedures have been developed with IR thermography. For example, Ref. [27] points out the use of IR cameras for inspecting wind turbine elements inside the nacelle (gearbox, generator, brake, etc.) or outside, such as the blades (ice detection). Due to the capability of near-surface defect detection, structure inspection is another field where IR image processing has gained attention [28]. In these applications, active IR thermography is the technique to detect flaws in concrete surfaces. A processing algorithm locates and quantifies the defect. The same procedure is explored by [29] for automated aerial inspection of aerospace structures. Specific algorithms were used to produce accurate results. Metallic corrosion [30], asphalt pavements [31], or even biomedical applications [32] are other examples of IR thermography use.

So, PV hot-spots can be precisely detected, isolated, and analyzed using IR inspection by well-trained maintenance teams, which has become a common practice in current PV applications, as shown in [33]. A thermal imaging camera captures thermal images of hot-spots that show a temperature distribution in terms of a colored pattern known as a thermogram. The following references discuss hot-spot observational analysis using thermal imaging and sometimes redundantly with electrical energy loss analysis due to hot-spots under shadowing in the photovoltaic solar array [34].

The design and development of hot-spot mitigation techniques using a simple, costless, and reliable method are proposed in [33,34]. The hot-spots analysis in the PV system was carried out using a FLIER i5 IRT.

Applying the above actions safeguards the PVM so a more detailed diagnostic can be done. In this sense, a set of references whose common denominator is IR thermography and red, green, and blue (RGB) cameras, using various techniques for specific analysis, are highlighted below. Most research [34,35,36,37,38] proposes IR imaging as the best technique to identify specific types of faults, including the development of hot-spots and areas, degradation effects, discolored PV cells, temperature degradation effects in bus bars, contact solder bonds, blisters, and broken solar cells with interrupted interconnects or cracks.

Drones make it possible to implement different configurations to detect different types of failures. One of the most used configurations is the dual configuration formed by an RGB camera and an IR thermographic camera [39]. Recent research [40,41,42,43,44] shows improvements in thermographic and artificial vision techniques oriented to PV diagnosis with drones. They develop algorithms to correct camera angles regarding the position of the panels in order to obtain more precise diagnostic data and find more exact locations of the defective PVMs among hundreds or thousands of PVMs in large PVIs.

Besides fault detection, condition monitoring is essential to keep the PV system fault free. The condition monitoring of the PV system has been approached with different techniques. One of the most used is the analysis of the I-V curve [45]. This technique compares the PV system’s normal working response represented by the I-V curve with the actual response to find deviations due to degradation. I-V curve monitoring gives essential information for monitoring, but it cannot isolate the cause of the degradation. A recent study [46] shows that I-V curves can detect most manufacturing defects but deeper analysis needs infrared images and electroluminescence (EL).

Imaging techniques (IR and EL) have been widely introduced in PV condition monitoring due to the advances in digital cameras [47]. As pointed out by [48], EL imaging involves high time for EL measurement, thus liming this technique to small-scale installations. Thermal cameras give surface temperature information through IR images [47]. Because it performs non-destructive tests, is portable, gives real-time information, and is a safety technology for humans, IR imaging is being adopted by many researchers [49].

One of the disadvantages [49] of IR imaging is the cost of the equipment. In [50], a drone-based IR thermography is proposed for PV plant monitoring. Although it is a low-cost approach, the solutions presented range from USD 6.000 to USD 15.000. Only the camera, like the ones used in [50,51] can cost more the USD 5.000. The increasing use of PV systems for power generation makes it necessary to develop low-cost approaches to protect even residential PV installations.

In this work, the development of a low-cost system prototype is proposed for the supervision of a medium or small photovoltaic installation based on the acquisition and treatment of thermographic images, with the aim of investigating the feasibility of a real implementation. The work focuses on the system’s ability to detect hot-spots in supervised panels and successfully report detected faults.

To achieve this goal, a low-cost thermal imaging camera is used for development, applying common image processing techniques, operating with OpenCV and MATLAB R2021b libraries. In this way, it is possible to demonstrate that it is achievable to successfully detect the hottest points of a PV installation with a much cheaper camera than the cameras used in today’s thermographic inspections, opening up the possibilities for the creation of a fully developed low-cost thermographic surveillance system.

This manuscript is organized as follows: In Section 2, sensors and devices used in the supervision of photovoltaic electrical energy are presented. The photovoltaic generation system used and the tests carried out are described in detail. The proposed method for PV supervision and predictive diagnosis to avoid failure occurrences is explained. In Section 3, the results of the experiments are presented. Section 4 contains a discussion of results as well as some additional considerations and findings obtained from the experimental trials. Finally, in Section 5, some conclusions regarding the advantages of the proposed method for fault prediction are drawn.

## 2. Materials and Methods

This section describes the choice of a thermographic camera suitable for the study, the preparation of an image acquisition setup, and the development of a method capable of processing these images for the localization of hot-spots on solar panels.

### 2.1. Materials

#### 2.1.1. Thermographic Camera

**Figure 1:**
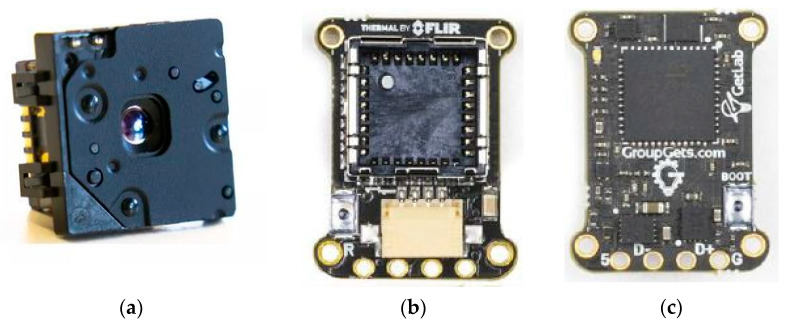
Modules chosen for the development of the low-cost hot-spot detection system. (**a**) Photograph of the FLIR Lepton 3.5 module. (**b**) PureThermal Mini Pro JST-SR Lepton Smart I/O (Socket, front view). (**c**) PureThermal Mini Pro JST-SR Lepton Smart I/O (CPU, rear view).

The camera chosen for the development of the work is the FLIR Lepton 3.5 thermal camera with FLIR Systems 3.2a radiometry (Figure 1). This small module was chosen first for its resolution and sensitivity. In addition, with its measuring range of −10 °C to 140 °C, it is sufficient for the detection of hot-spots, which at most can reach 120 °C, thus without giving up good thermal sensitivity. The normal operating temperature has also been a key point for the choice of this camera since the second option considered (the NF-522 of 200 × 150 px.) was not chosen because it has an operating temperature range of up to 45 °C, which, for outdoor work, without shade, can be risky. The major advantage of this module is its price with respect to its resolution and sensitivity, which the other options cannot beat. In addition, the 3.5 model with radiometry is able to detect the actual temperature of the readings and not an estimation based on the ambient temperature.

On the other hand, it should be noted that this module alone is incapable of transmitting information easily to, for example, a computer. This will require the purchase of an interface to enable communication. Various models from different manufacturers are available for the integration of the Lepton 3.5 module. For example, development boards with a socket compatible with Lepton 2.X and 3.5 are available.

The module chosen for the work is the PureThermal Mini Pro JST-SR Lepton Smart I/O Module developed by GroupGets. It is a programmable module for the chosen camera. It includes a serial configuration to easily operate on a host PC via UVC 1.0 USB, making use of a JST-SR to USB connection. It is compatible with Windows, Linux, Mac, and Android.

One of the reasons this module was chosen is that it offers access to in-house firmware development for custom applications. For Windows, there is the FLIR Lepton User application that supports radiometric readings from the camera for image acquisition.

The other features of the module are detailed below:

9 Hz color video transmission via USB in real-time.

STM32F412 ARM microprocessor, capable of internally processing images and transmitting them without the need for an external system.

Ability to upgrade the STM32 ARM MCU via USB. 5V power supply via USB.

Supports Lepton 2.X and 3.X modules.

Compact design. Dimensions: 19.5 × 15.32 mm.

The final purchase price, including taxes, duties, and shipping for the two modules is EUR 419.32.

#### 2.1.2. Photovoltaic Installation

**Figure 2:**
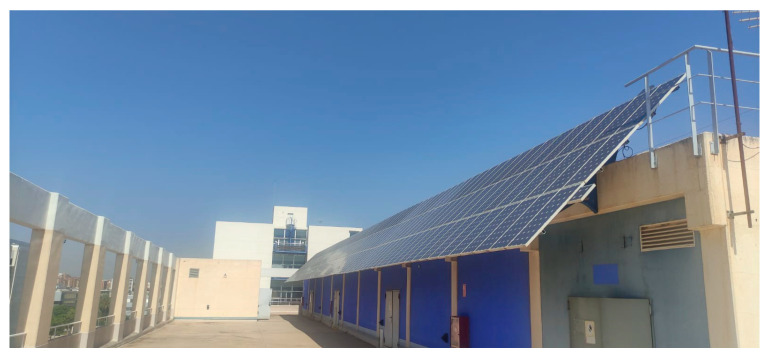
PV installation.

The tests are performed on a 17.5 kW photovoltaic installation located on the rooftop of the Escuela Técnica Superior de Ingeniería del Diseño (ETSID) at the Vera campus of the Universitat Politécnica de València (UPV) (Figure 2).

This is a grid-tied photovoltaic installation erected in 1999. It consists of its solar field, a connection system, six inverters that make up a balanced three-phase system, a protection system, and a data acquisition and transmission system for computer analysis.

The installation has a total of 234 Atersa A-75M panels. They are crystalline silicon modules of 21 V Voc and 17 V in the MPP. The rest of the characteristics are detailed in Table 1.

The panels of the field are arranged in a uniform plane at 45° on the horizontal plane, and the installation is electrically subdivided into seven solar fields. Solar field 1 consists of 42 panels connected to a direct current (DC) distribution board. Fields 2 to 7 are made up of 24 parallel branches of 8 panels in each series.

In the inverter room, there are six inverters (Tauro 4000/8), a DC distribution board, a display to control the voltage, current and power of each line of the installation, a protection board and a data acquisition system to process the information.

The entire plane on which the PV panels are located is elevated 3.23 m above the horizontal (from the ground to the lowest part of the panels). The orientation of the entire installation is south-southwest.

For testing purposes, the thermographic camera can be placed directly in front of the solar panels. There is a main area of approximately 7 × 32 m, and a more limited access area between the access to the terrace and the rest of the solar field of 2 × 12 m.

### 2.2. Methods

A method has been developed to achieve sufficient accuracy to detect hot-spots in the 160 × 120 px. resolution images obtained. To do this, first, all the captured images are conditioned by applying calibration corrections, adjusting the emissivity, and normalizing values. The next step is to correctly segment and automatically delimit the Region Of Interest (ROI) using only the thermal camera. Once the area where the hot-spot recognition is to be performed, the application for the detection and classification of the detected points is developed.

For this task, we have used MATLAB 2021b packages: Computer Vision Toolbox, Computer Vision Toolbox Interface for OpenCV in MATLAB, MATLAB Support Package for USB Webcams, and Statistics and Machine Learning Toolbox.

#### 2.2.1. Image Conditioning

Camera calibration is necessary for optical error compensation and distortion attenuation. For this task, MATLAB includes a very useful function for the calibration of any type of camera: the Camera Calibrator interface. Once this application is opened, it is necessary to enter the type of pattern to be recognized by the algorithm: “checkerboard”, “symmetric circle matrix” or “asymmetric circle matrix”. In this case, the first of the three options is selected, and the algorithm is informed of the size to expect the calibration pattern to be, in this case, 7 × 7 cm.

Once the type of pattern is selected, the images of the calibration set are loaded into the application, which are nothing more than thermographs of the real pattern taken from different distances and angles; and the program automatically detects the points of greatest contrast on the board. At this point, the calibration function can be executed, which generates a prediction of the appearance of these points in the images according to the calculations made from the pattern recognition. In this case, the application detected 13 of the 13 photos provided and reported that 2 of them were partially recognized. No image was automatically discarded. However, the application shows the errors between its prediction and the first detection and gives the option to remove the outliers from the computation. In this case, the two thermographs that added the most error were eliminated.

Once the calibration session is finished, a code is automatically generated with the optical parameters of the camera deduced by the application. This code is stored and can be used for image correction, even in real-time.

All thermal images have the defect of capturing not only the long-wave radiation emitted by a body but also the short and medium-length ones reflected and transmitted by the surrounding bodies. The emissivity correction in the PureThermal Mini Pro module is manual, i.e., it must be performed explicitly. This is why an emissivity correction must be performed, i.e., the error of the reading due to wave reception from other sources must be calculated. To do this, two thermographs of the same radiating body (any object) must be taken at two different temperatures. Through a series of calculations (Equation (1)), one can find the difference between the radiation emitted at the two temperatures and deduce what percentage of the radiation is due to the object and what percentage is not. Once this is known, a correction map, applicable to any image taken, is generated. In the case of correct emissivity correction, the temperature readings will be real since the radiation read will only be due to the emitting body.

(1) $$B=\frac{\left(A-\left(1-\frac{∆M}{∆t}\right)\cdot \frac{M_{t2}-\frac{∆M}{∆t}\cdot t_{2}}{1-\frac{∆M}{∆t}}\right)}{\frac{∆M}{∆t}}$$

where:

*A*: is the image to be corrected

*B*: is the corrected image

∆*M*: is the difference between the matrices of different temperatures (*M_t2_* − *M_t1_*)

∆*t*: is the temperature difference (*t_2_* − *t_1_*)

As mentioned above, RGB images have an 8-bit color depth, so each pixel takes a value from 0 to 255, depending on whether the radiation reaches it to a greater or lesser extent. This value is assigned to a color according to the palette selected in the Lepton User App. However, for RAW images, the color depth is 16-bit, which means that each pixel takes a value from 0 to 65,536. This generates a very low contrast image since, if there are no large temperature differences, most of the values will be concentrated near the median of its color map. Therefore, it is necessary to apply an Automatic Gain Control (AGC) or, failing that, a normalization of the RAW images.

If we import a .tiff file into MATLAB^®^, we see that its content exists, but the difference between the pixels of the matrix is very small in relation to its color depth, so its contrast is very small. To apply the normalization of the images, a small function (normGan.m) was developed. This function receives, as a parameter, the matrix T in ‘.tiff’ format and returns a normalized A matrix passed to grayscale (double values from 0 to 1), as well as the smallest and largest value of the non-normalized A matrix. For normalization, first, it divides each value of T by 65,536, and then it applies the normalization calculation expressed in Equation (2). It is important not to lose the matrix T since it is the only one that actually contains the thermal information.

(2) $$A_{norm}=\frac{A-a_{min}}{a_{max}-a_{min}}$$

**Figure 3:**
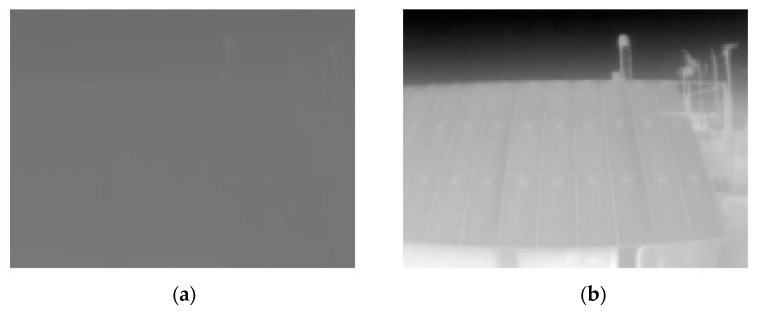
(**a**) Unnormalized RAW image; (**b**) resulting normalized image.

Thus, the color space is reduced to nothing more than what is necessary for each thermal image. This function will be called whenever it is necessary to normalize any RAW matrix. The contrast enhancement can be seen in Figure 3.

#### 2.2.2. Segmentation of the Region of Interest

The first step in any artificial vision application is, once the image pre-processing has been performed, to segment the area of interest to be analyzed so as to omit calculations in areas that do not require it. For this work, the part of interest is the area of the image where the solar panels are located. Once this area is detected, it is segmented and cropped, and then work on the search for the hot-spots in that area.

The method tested has been the application of an edge detection algorithm. These algorithms compare the value of the analyzed pixel with the values of neighboring pixels and binarizes their values, if the contrast between them is suficiently relevant. The way in which a pixel is compared with its neighbors is what differentiates some algorithms from others. In this work, two different detection algorithms were tested: “sobel” and “canny”.

The first one takes into account the direction of the gradient of the neighboring values once a Gaussian filter is applied, which results in a “blurred” or fuzzy image. Once this transformation is obtained, the algorithm binarizes the pixels where the grayscale gradient is larger. The second one operates in a very similar way, except that it additionally takes into account the gradient direction of its own neighbors: if both share the same direction, it is valued as a high contrast edge; if it does not share a gradient direction with any neighbor, it is considered a low contrast edge. If the analyzed pixel does not even represent a high point of the gradient, it is considered not to be an edge.

**Figure 4:**
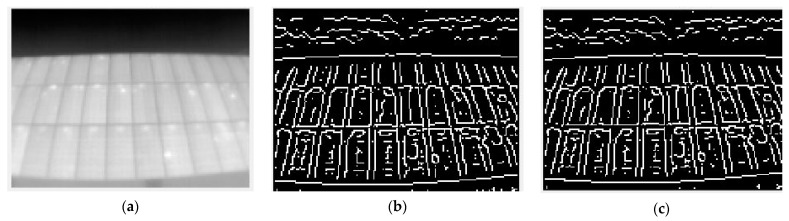
Different applications of edge detection filters for the same image. (**a**) Raw image; (**b**) Sobel filter; (**c**) Canny filter.

Applying these two filters to a normalized grayscale thermogram yields the results shown in Figure 4.

The best results are given for higher thresholds, i.e., more restrictive: they show only the detected edges of the higher gradient. It is observed how the “canny” filter obtains fewer details, i.e., performs a more demanding filtering. Therefore, for the following steps, the development will be done with this filter, since the ROI content is not of interest, only its detection, for the time being.

**Figure 5:**
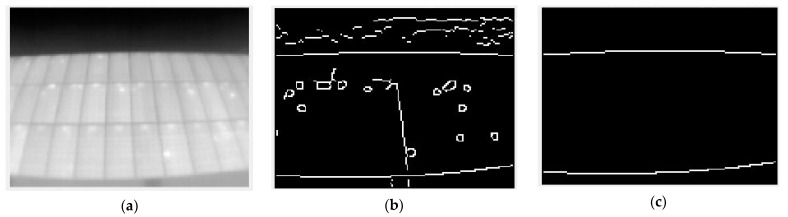
Result of removing small objects applied to the result of the “canny” filter. (**a**) Raw image; (**b**) Edge detection with Canny filter; and (**c**) Small objects removing.

The next step was to remove the smaller elements from the image treated with the “canny” filter. This is done with a simple function that removes those groups of linked pixels from a binary image that do not reach a given area (bwareaopen.m) in MATLAB. The result is shown in Figure 5.

**Figure 6:**
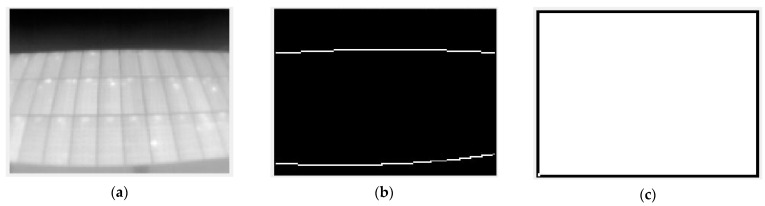
Result of filling the central area of the binary image. (**a**) Raw image; (**b**) Edges filtered; and (**c**) Area filled.

Once the result of the “canny” filter is cleaned, the solar field margins are obtained, as intended. A binary image is thus obtained, which is divided into three parts by the detected edges. If it were possible to invert the values of the central area, it would be possible to obtain a binary mask for the ROI segmentation. A simple solution is to make use of the imfill.m function in MATLAB. This function detects closed areas within a binary image and converts them from 0 to 1. To do this, the coordinates of the initial pixel from which to fill an area can be given as a parameter. Since all images are centered in the solar field, the central coordinate [60, 80] or [160/2, 120/2] will always lie within this region. Once the action is executed, the result is as shown in Figure 6.

**Figure 7:**
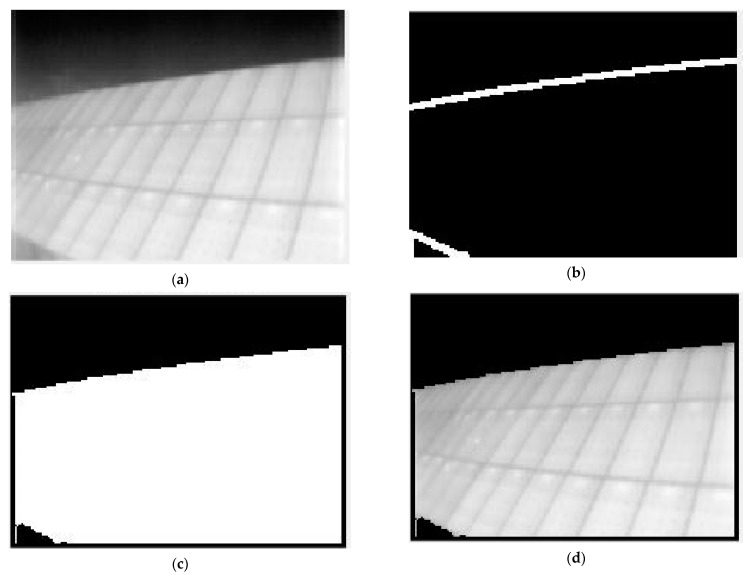
Example 1 of the final result of the automatic segmentation. (**a**) Raw image; (**b**) Edges thicked; (**c**) Area of interest; and (**d**) segmented image.

This result is due to the fact that the executed function has understood the whole image as a single open area since there are stretches of the edge lines that are so thin that it is considered an open gap. To solve this, a simple algorithm is applied to thicken the edges of a binary image, and the function imfill.m is called again. Next, the filled area is reduced with the same function used for the thickening to compensate for its effect and thus not use more pixels than necessary. Finally, it is filtrated to remove outlier pixels resulting from the transformation operations. The result is the correctly segmented area of interest. By performing an image multiplication, the original image segmented according to the desired ROI is obtained (Figure 7).

#### 2.2.3. Algorithm for Hot-Spot Detection

Once the area where the hot-spots are to be searched is segmented, an algorithm capable of labeling a certain number of hot-spots in an image and displaying their temperature is developed. For this purpose, a function is developed in MATLAB that will use the resources reviewed in previous sections for the conditioning, segmentation, and recognition of the thermographies made. The algorithm works in such a way that it always prioritizes the search for the hottest point, then keeps detecting points up to a user-specified amount. In addition to the number of detected points, the number of attempts for recognition and the limit temperature can also be configured, so that not only the hottest point can be displayed but all points below a threshold. Additionally, the physical characteristics of each detected hot-spot such as area, longest axis, shortest axis, and inclination with respect to the *X*-axis can be accessed.

Initially, the code executes all the image loading, correction, and processing instructions. Next, it segments the ROI and applies the search and labeling algorithm. The latter consists of two steps: the search and segmentation of the detected objects (hot-spots) and their subsequent labeling and calculation of their temperature.

The developed function receives as initial parameters:

- T: matrix containing the RAW information of the loaded image.
- n_points: number of hot-spots to be displayed. (Not to be confused with the number of hot-spots calculated. To display all detected hot-spots, the text ‘all’ is passed as a parameter).
- n_attempts: number of iterations that the function performs to detect the hottest points of the thermogram. The more attempts, the slower the algorithm becomes.
- window: size of the window of neighbors of the target pixel at each iteration. The larger the window, the more imprecise the search becomes and the faster the algorithm becomes; the opposite is true, if the window size decreases.
- t_lim: temperature limit for point detection. It sets the temperature from which the hot-spots are searched, e.g., if t_lim = 40, only the hottest spots in the image below 40 °C will be searched.

**Figure 8:**
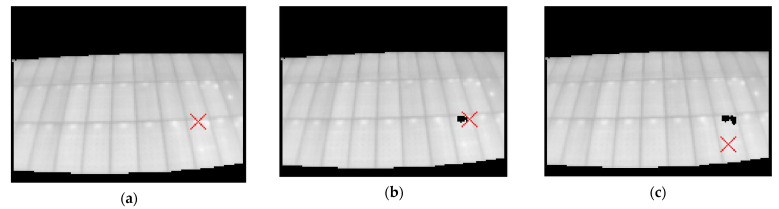
Visualization of the first three detected points. (**a**) First point; (**b**) Second point; (**c**) Third point.

**Figure 9:**
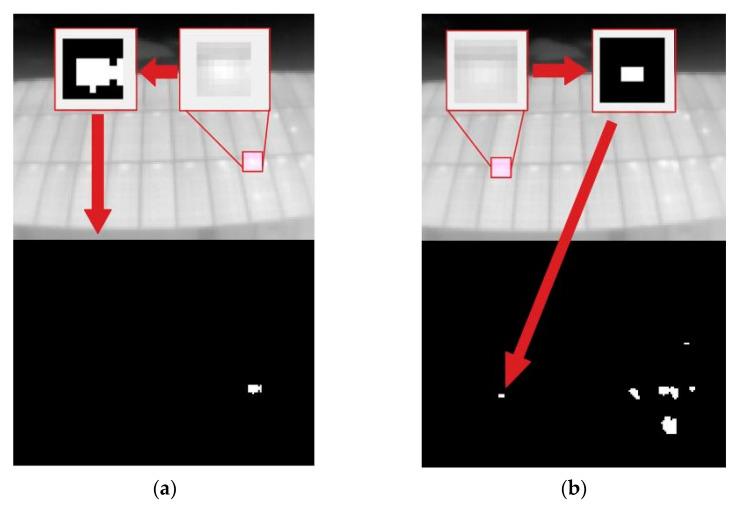
Binarization and storage of the neighboring pixel window. (**a**) Iteration 1; (**b**) Iteration 7.

The first step that the algorithm performs is to search for the hottest point of the image, and once it is found, is compared with the value of t_lim, and if it is lower than this, the position of the pixel is stored (Figure 8). Once the pixel position is obtained, a window (of variable size from the code) including the nearest neighbors is selected. The resulting matrix (n × n window, depending on the chosen size) is binarized with imbinarize.m with a high threshold (>0.9), thus obtaining the points of values closest to the selected pixel. The result of this binarization is stored in a binary image that will accumulate the results of each loop (Figure 9).

Finally, the segmented points are extracted from the original image, on which the loop is executed again. This ensures that the hottest point is not always detected but that the different points of the thermal image are selected in descending order at each iteration.

**Figure 10:**
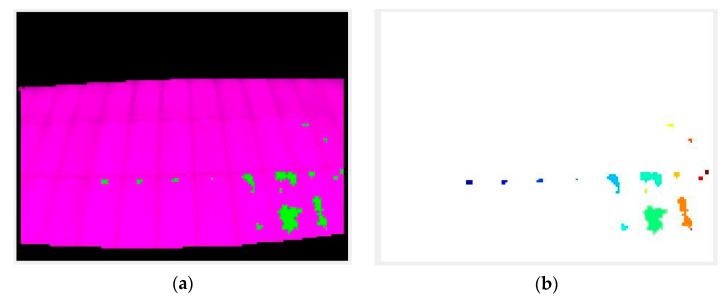
Result of the segmentation and labeling of the hot-spots. (**a**) Segmentation; (**b**) Neighboring.

Once the “attempts” indicated by the user are exhausted, the described loop ends, which results in a binary image with the detected zones. These zones can be overlapped without a problem and thus generate a larger object (Figure 10). The size of these is filtrated according to whether they are too small, to avoid noise and erroneous readings, or too large, since an area of the thermogram that is much more illuminated than the rest would end up being detected as a large hot-spot. These limit sizes are 2 pixels as the lower limit and 200 as the upper limit. It must be taken into account that the resolution of the images is 120 × 160, and a single solar cell never occupies more than 12 pixels. Objects of more than 200 pixels would mean more than 16 adjacent cells as hot-spots. By the very cause of the occurrence of hot-spots, it cannot happen that such a number of cells fall below the overall performance of the panel and thus increase their temperature simultaneously. It is for this reason that large detected objects are filtrated since their temperature, higher than that of the other points of the thermography, is probably the result of direct connections from the Sun or partial shading of the solar field.

Thus, with the binarized image of all localized hot-spots, each individual object (all the groups of pixels connected to each other) is labeled as a different hot-spot. For each point, its area, centroid, length, and width are obtained. Here, a further filtering is performed: any detected hot-spot, which is too long in relation to its width or too wide in relation to its length, is eliminated. This serves to filter possible edge readings of supports, buildings, or structures that may have passed through the first segmentation of the image and the subsequent filtering of objects by size.

To obtain the temperatures at which each of the points are located, previously, during the image processing, a call to the custom function calcTemp.m is made. This function receives an input parameter of a matrix (thermography), in RAW format of 120 × 160, and returns a corresponding matrix of 120 × 160, with the calculated temperature for each pixel of the image. In addition, it returns the maximum temperature, the minimum temperature, and the temperature gradient between these two values for the scale of the heat bar in the graphic. As mentioned in previous sections, the reading received by each pixel of the Lepton 3.5 operating in RAW, and with the “TLinear” function enabled, is translated to a 16-bit integer value. Each value represents the temperature in centikelvin (*cK*), so Equation (3) is applied to calculate its equivalence in Celsius degrees. Once the change of units has been applied, we have a matrix with the readings in degrees Celsius for each pixel.

(3) $$T_{C}=\frac{T_{cK}}{100}-273.15$$

where:

*T_C_*: is the calculated temperature in Celsius.

*T_cK_*: is the temperature in centikelvin of each pixel.

To find the temperature of each detected hot-spot, we simply perform an image multiplication of the obtained temperature matrix and the segmentation of the corresponding point. From the resulting image, the average of all non-zero pixels is calculated, and the pixel with the highest temperature is found. Thus, the average temperature of the hot-spot region (T_reg) and its maximum point temperature (T_punt) are obtained.

**Figure 11:**
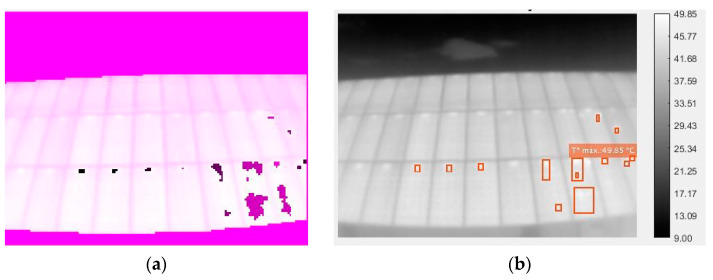
(**a**) segmentation of the hot-spots; (**b**) final image of the detected points, which are marked, and their calculated temperatures (only the highest temperature is shown for clarity reasons).

Finally, the original image is shown together with the locations obtained by the algorithm for each of the detected points. Each point also shows the average temperature of the contained region and the maximum point temperature in that region (Figure 11).

**Figure 12:**
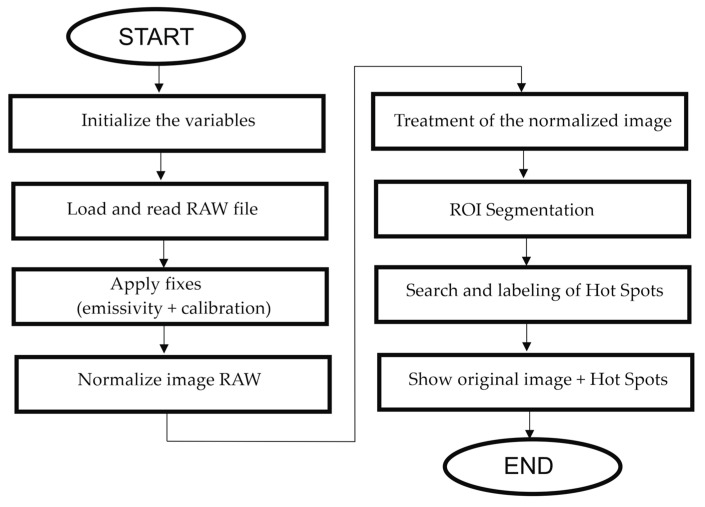
Global execution flux diagram of the hot-spot search and labelling algorithm.

**Figure 13:**
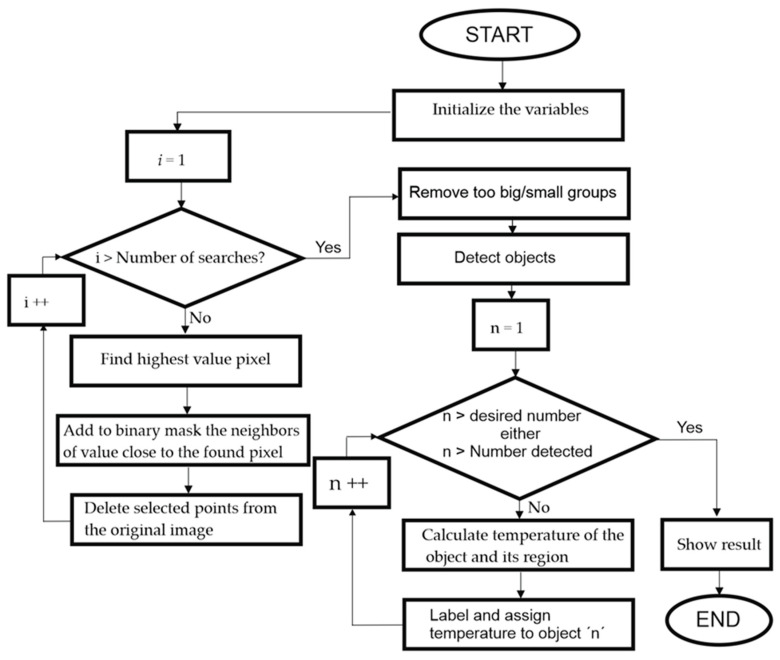
Diagram of execution of the hot-spot search and labelling algorithm.

Figure 12 and Figure 13 show the flow-diagram of the proposed method for hot-spot detection. The overall operation of the algorithm is described in Figure 12 and the search and labeling part is detailed in Figure 13.

## 3. Results

**Figure A1:**
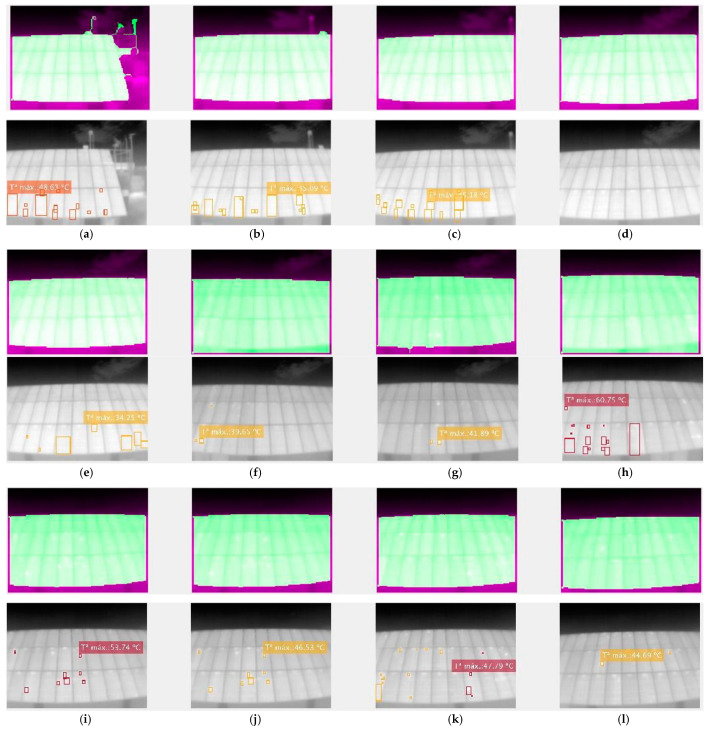

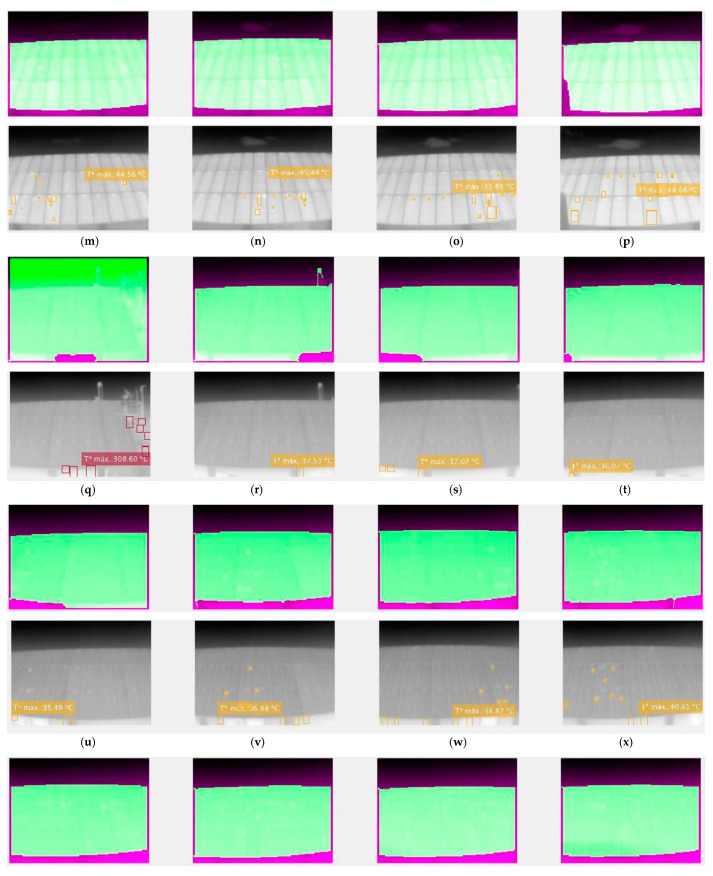

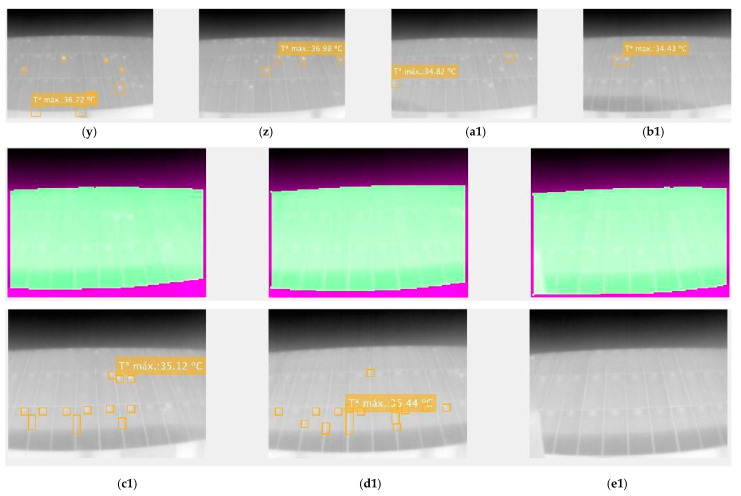
Images resulting from hot spot segmentation and labeling. A1(**a**–**p**) correspond to the thermographs taken at 16:00h (irradiance of 312.6 W/m^2^). A1(**q**–**e1**) correspond to those taken at 19:00h (irradiance of 2.81 W/m^2^).

The proposed hot-spot detection algorithm has been applied to a set of 31 images taken under the calculated optimal conditions: distance to the installation of 5 m, camera height 4.5 m, horizontal inspection angle 0°, and a variable position from 0 m to the end of the installation (32.4 m), at an interval of 2 m per thermography. Thus, the first 16 thermographs covering the total solar field voltage were taken at 16:00 h in the afternoon of 14 September 2022. Then, the same thermographs were taken again, under the same conditions, three and a half hours later, at 19:30 h on the same day. The results of these images, once segmented and treated, are presented in Figure A1, in Appendix A. As explained before, the first 16 correspond to the thermographs taken at 16:00h (irradiance of 312.6 W/m^2^) and the others to those taken at 19:00h (irradiance of 2.81 W/m^2^).

For all of them, the algorithm was used to ask for the return of all the hot-spots it was able to find. The parameters of the function are set to: input matrix Tn; hot-spots: ‘all’; number of attempts: 100; window size: 3; and temperature limit: 30.

A large hit rate is observed for the first 16 thermographs, which is not repeated for the next 15. This can be explained because the irradiance at 19:00 is very low (<50 W/m^2^). For these irradiances, the temperature readings are not reliable since the thermal contrast for such irradiances is very small. Moreover, because the solar cells produce less current, defective cells that have reached reverse polarization do not end up dissipating as much power. Thus, it can be seen how from Figure A1(m) onwards in the image set, the artifacts and outliers start to become constant. However, due to the poor state of the PV installation, it can be seen how, even with so little solar radiation, visible hot-spots are produced along the entire solar field. Even so, the algorithm is still able to detect these hot-spots.

For the first 16 images, the segmentation is successful and does not fail. For the next 16, because of the low thermal contrast, the segmented area is confused with the surroundings of the installation, thus giving rise to readings of hot-spots outside the solar field. Obviously, these readings are erroneous; even if the temperature readings are correct, it is of no interest to know the values at these points.

The maximum stored temperatures are listed in Table 2.

The average temperature of the maximum hot-spots for the images taken at 16:00 is 43.04 °C. The average temperature of the maximum hot-spots for the images taken at 19:30 is 35.36 °C (excluding temperature data from outside the PV field). If the normal operating temperature of the cells is calculated according to Equations (4) and (5) taken from the Tamizh-Mani method, under the meteorological conditions taken at the two times of the day, it can be calculated that the hot-spots during the hours with the highest irradiance exceed the normal operating temperature by 52.3%, and during the hours with the lowest irradiance by 53.53%.

T_16:00_ = 0.943 × 28.8 + 0.028 × 312.6 − 1.528 × 11.6 + 4.328 = 22.51 °C (4)

T_19:00_ = 0.943 × 26.1 + 0.028 × 2.81 − 1.528 × 6.6 + 4.328 = 18.93 °C (5)

There are two specific cases in which the algorithm does not detect anything or only detects one point, although it is obvious that there are others. These are the case of images 31 and 12, respectively. In the case of Figure A1(e1), the unusual response is attributed to the appearance in the lower left corner of a piece of the building itself, which, being hotter than the solar field, will have accumulated all the loop attempts in that area and then the resulting object will have been filtered out because it exceeds the maximum size of the client point. The case of Figure A1(l) is due to an exceptional case where the view window encloses only very-high intensity points. Once this area is binarized, as all-high-value dots remain in the same window, the binarization threshold, which is set to 0.9, determines that there is not enough contrast between the dots, and therefore the whole window is set to 0. This implies that no pixels are stored in the binary hot-spot mapping, and therefore, no such pixels are removed from the original image so that in the next iteration, the same hot-spot is still detected over and over again. This problem can be solved either by increasing the size of the window so that the contrast with the other neighbors becomes noticeable or by changing the threshold value of the window binarization to a lower one.

**Figure 14:**
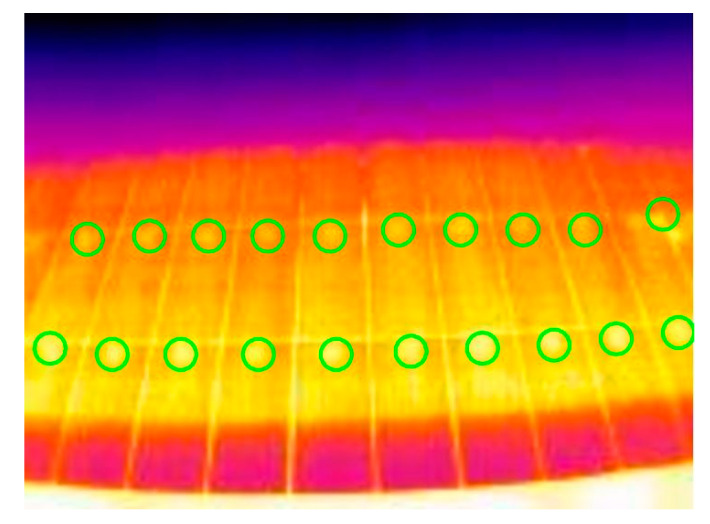
Image with the hot-spots per junction box highlighted in green circles.

Already at first glance, the thermographs show a key flaw in the installation: the junction boxes are affecting the temperature of the plate. This can be seen in the fact that each of the modules has, without exception, an area of constant higher temperature and size between the different plates just at the upper edge of the frame (Figure 14).

**Figure 15:**
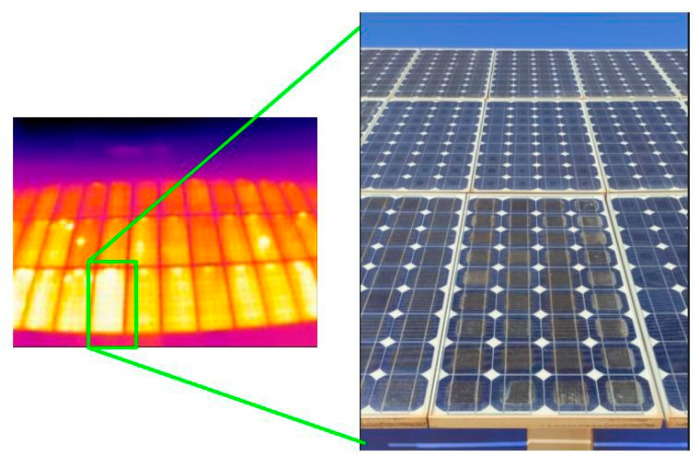
Image with a damaged PV panel.

However, this is not the major flaw detected in the installation. The first time thermography of the solar field was taken, it was very clear that there were multiple hot-spots throughout the entire installation. Some of these points were not cells but the entire PV panels (Figure 15). The damage to the installation is multiple, and the wear of the components is, in some cases, extreme. Ethylene Vinyl Acetate (EVA) wear does not always have to mean hot-spots, but by comparing the thermal images with the damaged panels, it is clear what is causing all these hot-spots to appear.

It can be concluded that the condition of the installation is bad, and it generates constant performance losses. Urgent intervention is needed to repair or replace modules, or soon more panels will be irreversibly damaged. Some panels from which normal temperature readings are obtained do not show such severe degradation.

**Figure 16:**
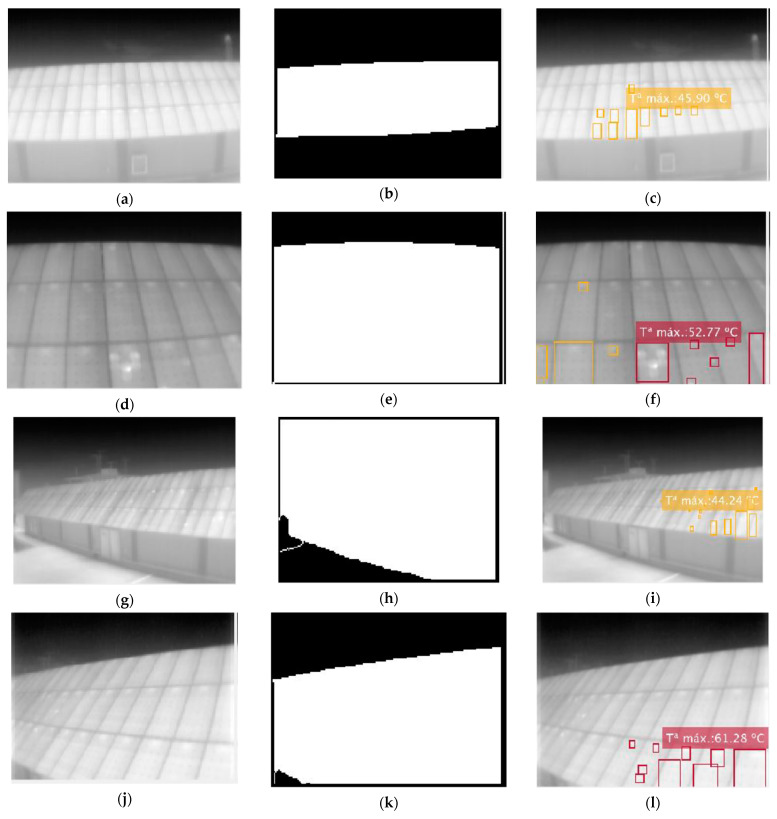
Different thermographies maintaining position and angle for distance comparison. (**a**,**d**,**g**,**j**) Raw images; (**b**,**e**,**h**,**k**) segmented images; and (**c**,**f**,**i**,**l**) final image.

For the distance evaluation, the algorithm is asked to display all the hot-spots it finds in images with the same angle, position, and height (Parameters: T,’all’,100,2). The modified parameter, in this case, is the distance to the facility. Thus, Figure 16a–f show the same area of the facility only at distances of 7 and 3 m, respectively. Figure 16g–l follows the same criterion but with a steeper angle than the previous ones.

The first difference observed is the change in temperature reading. In Figure 16, only the maximum point temperature of the hottest point is shown. It can be seen how, for the thermographs closer to the installation, a much higher temperature is detected than the one read at a greater distance. It is worth noting that for the case of Figure 16j–l it can be expected that such high-temperature points are the result of the reflection of the Sun on the panels. However, for Figure 16a–f, it becomes evident that for the longer distance image, the temperatures are much lower than what might be expected. One possible justification for this is that at large distances, the value that each camera pixel records is a “blurred” image of the region. This effect can lead to a weighting of the temperature at that point and thus obtain a lower temperature than that read from close-up.

Therefore, it can be stated that at a greater distance, there is no loss of effectiveness in recognizing the area of interest, but the temperature measurement is impaired due to the low geometric resolution.

**Figure 17:**
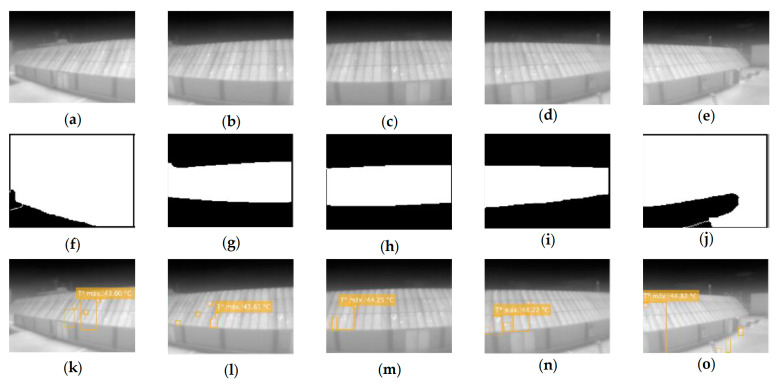
Variation of the horizontal angle (−35°, −15°, 0°, 15°, 35°) for the distance of 7 m. (**a**–**e**) Raw images; (**f**–**j**) segmented images; (**k**–**o**) final images.

The previous process is repeated for the comparison of the results according to the horizontal angle. To do this, thermographs will be taken from the same distance, position and height, and the angle will be varied (Figure 17).

It can be seen that for less steep angles, the area of interest detection algorithm works much better than for larger angles. This is due, once again, to the low resolution of the camera: at longer distances, the contrast is much lower, so the edge search is much more complicated, and artifacts end up appearing that lead to the selection of an unwanted area of the image.

**Figure 18:**
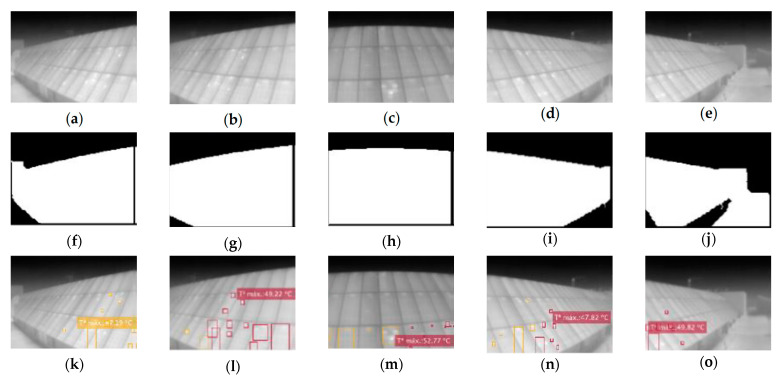
Variation of the horizontal angle (−35°, −15°, 0°, 15°, 35°) for the distance of 3 m. (**a**–**e**) Raw images; (**f**–**j**) segmented images; (**k**–**o**) final images.

Another observation that can be extracted from this sample is that, despite detecting the ROI correctly, in the set for 7 m (Figure 17), the hot-spots are not detected correctly, while in the same case, in the set for 3 m, they are. This is in agreement with the results discussed in the previous section. Focusing on the segmentation results for the 3-m set, we see that, although detection fails, the failure is less critical than in the 7-m case (Figure 18).

## 4. Discussion

The feasibility of using a low-cost camera to automatically detect hot-spots in PV installations has been demonstrated. The use of the FLIR Lepton 3.5 camera, together with the PureThermal Mini Pro module, has been satisfactory from this perspective. Considering that this is a development module, and not a fully functional thermal camera, the smart I/O adapter module, together with the specific user application, is very useful as an interface between a common computer and the hardware module. Still, far from being an advanced interface, the Lepton User App allows important operations to be performed on the camera without accessing its embedded software, such as Flat Field Correction (FFC) and RAW or RGB operation.

The temperature measurement range has been adequate for the application developed, not having recorded temperatures that exceeded any of the specified limits (−10 °C to 140 °C).

As for the spatial resolution of 120 × 160 pixels, it has proved to be sufficient to successfully detect the hottest points of the installation under study. Although, it is not the most desirable resolution for the purpose, since in the field of automatic detection, it has proved difficult to select precise areas and recognize the smallest points.

**Figure 19:**
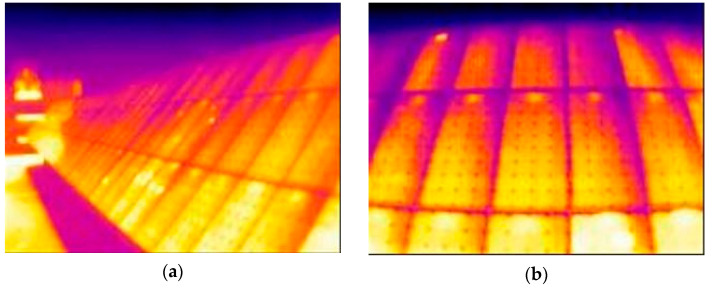
Sample distance-resolution-distortion relationship based on scene size. (**a**) Large scene image and low distortion. (**b**) Small scene image and high distortion.

The field of view of 57° horizontally and 71° diagonally has proven to be more of a problem than an advantage for the intended application, as its relationship to spatial resolution brings with it certain disadvantages. Despite being able to take thermal measurements of very large scenes, its limited resolution makes these measurements at the extremes of the images very inaccurate and, therefore, not very reliable (Figure 19a). On the other hand, if more precise measurements are to be made by bringing the camera closer to its target, the “fisheye” effect is substantially increased (Figure 19b).

The physical characteristics of the camera are a clear advantage for the required application. The weight and size of the camera, together with the module (19.5 × 15.32 mm, <200 gr.), makes it very easy to handle and allows it to be placed in virtually any configuration required. All this is especially useful for the inspection of PV installations, as considerable heights are usually required for good thermographic readings. A high weight at the required heights can cause problems. In addition, its small size generates very low wind resistance, so image stability can be assured with even the most rudimentary system.

Finally, the communication and power supply via the Smart I/O module via JST-USB is very convenient. With a power supply voltage of 5 V and a current consumption of 30 mA in normal operation and 130 mA when using the shutter, its power consumption never exceeds 650 mW of power. This means that it can be powered via USB without any problem and opens the door to its use with light batteries. In addition, being a programmable module, not only can it communicate via USB, but the module can also be reprogrammed via USB.

The extracted image banks have proved to be sufficient for testing the hot-spot detection rate of the installation. However, not all the images taken have been satisfactorily analyzed; many of them contain readings that may give diagnostic errors.

**Figure 20:**
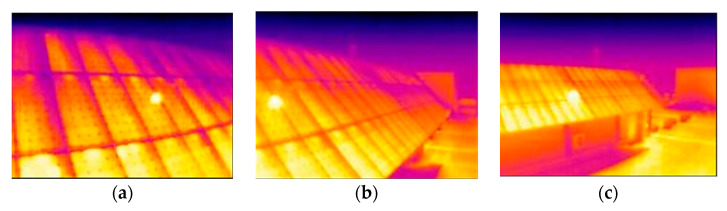
Three thermographs affected by the reflection of the Sun. (**a**) 5 August 11:29, dist.: 3 m, angle: 20°; (**b**) 5 August 11:37, dist.: 3 m, angle: 45°; and (**c**) 5 August 11:39, dist.: 7 m, angle: 45°.

The main reason why misdiagnosis occurs is due to the presence of thermal reflection, mainly from the Sun. Figure 20 shows how, when the Sun strikes the plates at just the right angle, the thermography clearly shows reflections in the solar panels.

**Figure 21:**
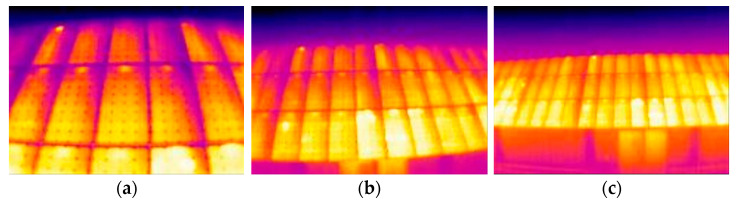
Comparison to show the uniformity of the thermal reading at the limiting and calculated distances. (**a**) 3 m away; (**b**) 5 m away; and (**c**) 7 m away.

The height of the camera was adjusted to the minimum calculated value; therefore, the height at which it was finally placed (4.5 m) is not the optimum (5 m). This is evident in the thermo-graphs taken at the calculated ideal distance (6 m from the installation) when a uniform reading of the temperatures of all the plates is not obtained (Figure 21b). This effect is even more pronounced when the distance is smaller (Figure 21a).

However, by using a greater height, this effect would be reduced. In fact, when the camera is moved further away, there is an improvement in the uniformity of the solar field readings (Figure 21c) since at a greater distance, the equation used to determine the optimum height would result in a lower minimum height.

On the other hand, it is safe to say that the time of day greatly affects the readings. In addition to the possible variations due to the position of the Sun, the irradiance received by the solar field and the ambient temperature vary the results of the thermographs. These vary according to the time of day, being higher at noon and lower in the morning and evening.

This work aims to lay the groundwork for the development of a fully autonomous hot-spot recognition system. The proposal reviewed in this work has demonstrated the feasibility, under the conditions reviewed, of using the Lepton 3.5 camera for its application in the monitoring of photovoltaic installations. To take full advantage of the potential of this piece of hardware, new commands can be programmed through its flash memory and thus implement an algorithm similar to the one developed in this work autonomously, without the need for an external PC. With this, a whole embedded and autonomous system for hot-spot recognition could be integrated.

The main points to be further developed in this line of work would be the addition of a low-cost RGB camera to improve the recognition and location of the points; an improvement of the assembly that would allow the thermal module to perform sweeps in the X and Y axes, or even raise and retract the support mast; the programming of the algo-rhythms in the same module or the application in real-time of the analysis (video transmission).

## 5. Conclusions

The programming of an external algorithm for processing the acquired thermal images has made it possible to test the capabilities of the Lepton 3.5 module and the PureThermal Mini Pro interface for detecting hot-spots in a medium-sized photovoltaic installation. In doing so, the main premise of this work has been achieved: to acquire a thermal camera that would fit the purpose without exceeding the 500 euro limit. The final prototype developed could be purchased for a total of 479.42 euros, being 419.32 euros the full price of the thermal module.

As could be seen, at distances of between 3 and 5 m, the module’s performance is sufficient to detect and label hot-spots on a medium-sized installation. At greater distances, the resolution becomes insufficient to accurately detect the smallest hot-spots.

Since this is an iterative algorithm, its execution time consumption depends directly on the number of attempts. The higher this value, the longer it will take the algorithm to finalize the search. The best-performing readings have been seen on thermographs closest to the plates. Images taken at a 3 m distance have the most accurate locations of hot-spots and the most reliable temperature readings.

All in all, it can be concluded that the Lepton 3.5 camera has the ability to locate and label hot-spots in a PV installation with certain limitations. These can be reduced to:

- The perpendicular distance to the installation must not exceed 5 m from the point of camera placement to the nearest panel of the installation.
- The horizontal angle of inspection may not be greater than ±20° at distances of 5 m and may be extended if the distance to the installation is less.
- Measurements should be carried out with irradiances greater than 600 W/m^2^ so that the thermal contrast is sufficient.

Finally, during the discussion of the results, it has been possible to diagnose the state of the PV plant under study. It has become clear that it presents a very damaged state and that its solar production will be seriously compromised. The greatest damage can be seen in the two lowest fila of the installation, where the degradation of the EAV is not only detected by infrared thermography, but it is also possible to observe this degradation visually. Some hot-spots detected reach temperatures of 65°, causing irreversible damage to the plates. Bearing in mind that this is a 1999 installation that has not received replacements since then, one can understand the state it is in, as the solar panels are in their last years of useful life. The damage to the system is severe and highlights the importance of proper maintenance. However, thanks to this, it has been possible to test the camera under real hot-spot conditions.

## Figures and Tables

**Table 1:** Characteristics of Atersa A-75M panels.

Electrical Characteristics	Value	Units
Power	75	W
N^o^ of cells	36	−
Maximum Power Point Current (*I_MPP_*)	4.40	A
Maximum Power Point Voltage (*V_MPP_*)	17.00	V
Short Circuit Current (*I_sc_*)	4.80	A
Open Circuit Voltage (*V_o_c*)	21	V
Temperature Coeficient of *I* (*α*)	2	mA/°C
Temperature Coeficient of *V* (*β*)	−97.20	mV/°C
Maximum System Voltage	700	V
PHYSICAL CHARACTERISTICS	Value	Units
Dimensions	1200 × 527 × 35	mm
Weight (approx.)	7.50	kg

**Table 2:** Maximum temperature recorded in each of the thermographs.

Image	Temp. (°C)	Image	Temp. (°C)
1	48.63	17	-
2	45.09	18	-
3	45.18	19	-
4	-	20	-
5	34.25	21	-
6	39.65	22	-
7	41.89	23	-
8	60.75	24	-
9	53.74	25	-
10	46.53	26	36.98
11	47.79	27	34.82
12	44.69	28	34.43
13	44.56	29	35.12
14	45.44	30	35.44
15	45.85	31	-
16	44.66	-	-

## Data Availability

Not applicable.

## Appendix A

This appendix shows the results of the experiments presented in the Section 3.

## References

[1] García E., Ponluisa N., Quiles E., Zotovic-Stanisic R., Gutiérrez S.C. (2022). Solar Panels String Predictive and Parametric Fault Diagnosis Using Low-Cost Sensors. Sensors.

[2] García E., Quiles E., Correcher A., Morant F. (2022). Predictive Diagnosis Based on Predictor Symptoms for Isolated Photovoltaic Systems Using MPPT Charge Regulators. Sensors.

[3] García E., Quiles E., Zotovic-stanisic R., Gutiérrez S.C. (2022). Predictive Fault Diagnosis for Ship Photovoltaic Modules Systems Applications. Sensors.

[4] Livera A., Theristis M., Makrides G., Georghiou G.E. (2019). Recent Advances in Failure Diagnosis Techniques Based on Performance Data Analysis for Grid-Connected Photovoltaic Systems. Renew Energy.

[5] Ferrara C., Philipp D. (2012). Why Do PV Modules Fail?. Energy Procedia.

[6] Santhakumari M., Sagar N. (2019). A Review of the Environmental Factors Degrading the Performance of Silicon Wafer-Based Photovoltaic Modules: Failure Detection Methods and Essential Mitigation Techniques. Renew. Sustain. Energy Rev..

[7] International Electrotechnical Commission (2021). Terrestrial Photovoltaic (PV) Modules—Design Qualification and Type Approval.

[8] Tanesab J., Parlevliet D., Whale J., Urmee T., Pryor T. (2015). The Contribution of Dust to Performance Degradation of PV Modules in a Temperate Climate Zone. Solar Energy.

[9] Kazem H.A., Chaichan M.T. (2019). The Effect of Dust Accumulation and Cleaning Methods on PV Panels’ Outcomes Based on an Experimental Study of Six Locations in Northern Oman. Solar Energy.

[10] Kimsong S., Kaneko T., Hara Y., Masuda A., Isomura M. Effect of High Impulse Voltage on Potential Induced Degradation in Crystalline Silicon Photovoltaic Modules. Proceedings of the AM-FPD 2018—25th International Workshop on Active-Matrix Flatpanel Displays and Devices: TFT Technologies and FPD Materials.

[11] Sharma S., Raina G., Malik P., Sharma V., Sinha S., Sharma S., Raina G., Sinha S., Malik P., Sharma V. (2022). Different Degradation Modes of PV Modules: An Overview. Adv. Nanotechnol. Energy Environ..

[12] Hsu S.T., Long Y.S., Ma H.C. (2015). Vibration Influence for Transporting Photovoltaic Cell. Appl. Mech. Mater..

[13] Oliveira M.C.C.d., Diniz Cardoso A.S.A., Viana M.M., Lins V.d.F.C. (2018). The Causes and Effects of Degradation of Encapsulant Ethylene Vinyl Acetate Copolymer (EVA) in Crystalline Silicon Photovoltaic Modules: A Review. Renew. Sustain. Energy Rev..

[14] Janssen G.J.M., Stodolny M.K., van Aken B.B., Loffler J., Lamers M.W.P.E., Tool K.J.J., Romijn I.G. (2019). Minimizing the Polarization-Type Potential-Induced Degradation in PV Modules by Modification of the Dielectric Antireflection and Passivation Stack. IEEE J. Photovolt..

[15] Hetita I., Zalhaf A.S., Mansour D.E.A., Han Y., Yang P., Wang C. (2022). Modeling and Protection of Photovoltaic Systems during Lightning Strikes: A Review. Renew. Energy.

[16] Sun G., Tu X., Wang R. (2019). Research on the Potential-Induced Degradation (PID) of PV Modules Running in Two Typical Climate Regions. Clean Energy.

[17] Blake F.A., Hanson K.L. The Hot-Spot Failure Mode for Solar Arrays. Proceedings of the 4th Intersociety Energy Conversion Engineering Conference.

[18] Ali M.U., Saleem S., Masood H., Kallu K.D., Masud M., Alvi M.J., Zafar A. (2022). Early Hotspot Detection in Photovoltaic Modules Using Color Image Descriptors: An Infrared Thermography Study. Int. J. Energy Res..

[19] Tang S., Xing Y., Chen L., Song X., Yao F. (2021). Review and a Novel Strategy for Mitigating Hot Spot of PV Panels. Solar Energy.

[20] Hajjaj C., Bouaichi A., Zitouni H., Alami Merrouni A., Ghennioui A., Ikken B., Benhmida M., Choukri M., Regragui M. (2020). Degradation and Performance Analysis of a Monocrystalline PV System without EVA Encapsulating in Semi-Arid Climate. Heliyon.

[21] Dhimish M., Holmes V., Mehrdadi B., Dales M. (2017). The Impact of Cracks on Photovoltaic Power Performance. J. Sci. Adv. Mater. Devices.

[22] Li B., Delpha C., Diallo D., Migan-Dubois A. (2021). Application of Artificial Neural Networks to Photovoltaic Fault Detection and Diagnosis: A Review. Renew. Sustain. Energy Rev..

[23] Pendem S.R., Mikkili S. (2018). Modelling and Performance Assessment of PV Array Topologies under Partial Shading Conditions to Mitigate the Mismatching Power Losses. Solar Energy.

[24] Matusz-Kalasz D., Bodnar I. Monitoring and Diagnostics of Photovoltaic Cells by Electroluminescence. Proceedings of the 2022 23rd International Carpathian Control Conference, ICCC 2022.

[25] Muñoz J., Lorenzo E., Martínez-Moreno F., Marroyo L., García M. (2008). An Investigation into Hot-Spots in Two Large Grid-Connected PV Plants. Prog. Photovolt. Res. Appl..

[26] Qu Z., Jiang P., Zhang W. (2020). Development and Application of Infrared Thermography Non-Destructive Testing Techniques. Sensors.

[27] Civera M., Surace C. (2022). Non-Destructive Techniques for the Condition and Structural Health Monitoring of Wind Turbines: A Literature Review of the Last 20 Years. Sensors.

[28] Yumnam M., Gupta H., Ghosh D., Jaganathan J. (2021). Inspection of Concrete Structures Externally Reinforced with FRP Composites Using Active Infrared Thermography: A Review. Constr. Build. Mater..

[29] Deane S., Avdelidis N.P., Ibarra-Castanedo C., Zhang H., Yazdani Nezhad H., Williamson A.A., Mackley T., Davis M.J., Maldague X., Tsourdos A. (2019). Application of NDT Thermographic Imaging of Aerospace Structures. Infrared. Phys. Technol..

[30] Doshvarpassand S., Wu C., Wang X. (2019). An Overview of Corrosion Defect Characterization Using Active Infrared Thermography. Infrared. Phys. Technol..

[31] Vyas V., Patil V.J., Singh A.P., Srivastava A. (2019). Application of Infrared Thermography for Debonding Detection in Asphalt Pavements. J. Civ. Struct. Health Monit..

[32] Magalhaes C., Mendes J., Vardasca R. (2021). Meta-Analysis and Systematic Review of the Application of Machine Learning Classifiers in Biomedical Applications of Infrared Thermography. Appl. Sci..

[33] Dhimish M., Holmes V., Mehrdadi B., Dales M., Mather P. (2017). Photovoltaic Fault Detection Algorithm Based on Theoretical Curves Modelling and Fuzzy Classification System. Energy.

[34] Chaudhary A.S., Chaturvedi D.K. (2017). Observing Hotspots and Power Loss in Solar Photovoltaic Array Under Shading Effects Using Thermal Imaging Camera. Int. J. Electr. Mach. Drives.

[35] Ahmed W., Hanif A., Kallu K.D., Kouzani A.Z., Ali M.U., Zafar A. (2021). Photovoltaic Panels Classification Using Isolated and Transfer Learned Deep Neural Models Using Infrared Thermographic Images. Sensors.

[36] Ali M.U., Khan H.F., Masud M., Kallu K.D., Zafar A. (2020). A Machine Learning Framework to Identify the Hotspot in Photovoltaic Module Using Infrared Thermography. Solar Energy.

[37] Du B., He Y., He Y., Zhang C. (2020). Progress and Trends in Fault Diagnosis for Renewable and Sustainable Energy System Based on Infrared Thermography: A Review. Infrared. Phys. Technol..

[38] Huerta Herraiz Á., Pliego Marugán A., García Márquez F.P. (2020). Photovoltaic Plant Condition Monitoring Using Thermal Images Analysis by Convolutional Neural Network-Based Structure. Renew. Energy.

[39] Henry C., Poudel S., Lee S.W., Jeong H. (2020). Automatic Detection System of Deteriorated PV Modules Using Drone with Thermal Camera. Appl. Sci..

[40] Liao K.C., Lu J.H. (2020). Using Matlab Real-Time Image Analysis for Solar Panel Fault Detection with UAV. J. Phys. Conf. Ser..

[41] Wang J., Weng H., Yan Y., Zimmerman S., Abdelkefi A., Park J., Lee D. (2019). Precise Inspection Method of Solar Photovoltaic Panel Using Optical and Thermal Infrared Sensor Image Taken by Drones. IOP Conf. Ser. Mater. Sci. Eng..

[42] Lee D.H., Park J.H. (2019). Developing Inspection Methodology of Solar Energy Plants by Thermal Infrared Sensor on Board Unmanned Aerial Vehicles. Energies.

[43] Alsafasfeh M., Abdel-Qader I., Bazuin B., Alsafasfeh Q., Su W. (2018). Unsupervised Fault Detection and Analysis for Large Photovoltaic Systems Using Drones and Machine Vision. Energies.

[44] López-Fernández L., Lagüela S., Fernández J., González-Aguilera D. (2017). Automatic Evaluation of Photovoltaic Power Stations from High-Density RGB-T 3D Point Clouds. Remote Sens..

[45] Fadhel S., Delpha C., Diallo D., Bahri I., Migan A., Trabelsi M., Mimouni M.F. (2019). PV Shading Fault Detection and Classification Based on I-V Curve Using Principal Component Analysis: Application to Isolated PV System. Solar Energy.

[46] Gallardo-Saavedra S., Hernández-Callejo L., Alonso-García M.d.C., Santos J.D., Morales-Aragonés J.I., Alonso-Gómez V., Moretón-Fernández Á., González-Rebollo M.Á., Martínez-Sacristán O. (2020). Nondestructive Characterization of Solar PV Cells Defects by Means of Electroluminescence, Infrared Thermography, I–V Curves and Visual Tests: Experimental Study and Comparison. Energy.

[47] Kandeal A.W., Elkadeem M.R., Kumar Thakur A., Abdelaziz G.B., Sathyamurthy R., Kabeel A.E., Yang N., Sharshir S.W. (2021). Infrared Thermography-Based Condition Monitoring of Solar Photovoltaic Systems: A Mini Review of Recent Advances. Solar Energy.

[48] Waqar Akram M., Li G., Jin Y., Chen X., Zhu C., Zhao X., Aleem M., Ahmad A. (2019). Improved Outdoor Thermography and Processing of Infrared Images for Defect Detection in PV Modules. Solar Energy.

[49] Herraiz Á.H., Marugán A.P., Márquez F.P.G. (2020). A Review on Condition Monitoring System for Solar Plants Based on Thermography. Non-Destr. Test. Cond. Monit. Tech. Renew. Energy Ind. Assets.

[50] Kirsten Vidal de Oliveira A., Aghaei M., Rüther R. (2020). Aerial Infrared Thermography for Low-Cost and Fast Fault Detection in Utility-Scale PV Power Plants. Solar Energy.

[51] Teubner J., Buerhop C., Pickel T., Hauch J., Camus C., Brabec C.J. (2019). Quantitative Assessment of the Power Loss of Silicon PV Modules by IR Thermography and Its Dependence on Data-Filtering Criteria. Prog. Photovolt. Res. Appl..

